# MiR-181c-5p Promotes Inflammatory Response during Hypoxia/Reoxygenation Injury by Downregulating Protein Tyrosine Phosphatase Nonreceptor Type 4 in H9C2 Cardiomyocytes

**DOI:** 10.1155/2020/7913418

**Published:** 2020-07-25

**Authors:** Sheng Wang, Liang Ge, Dengwen Zhang, Lin Wang, Hao Liu, Xiaodong Ye, Wanling Liang, Jun Li, Haichun Ma, Yin Cai, Zhengyuan Xia

**Affiliations:** ^1^Department of Anesthesiology, Guangdong Cardiovascular Institute, Guangdong Provincial People's Hospital, Guangdong Academy of Medical Sciences, Guangdong, China; ^2^Department of Anesthesiology, The University of Hong Kong, Hong Kong SAR, China; ^3^Department of Anesthesiology, The First Hospital, Jilin University, Jilin, China; ^4^Department of Cardiology, The Second Affiliated Hospital of Guangzhou Medical University, Guangzhou Institute of Cardiovascular Disease, Guangdong, China; ^5^Department of Pharmacology and Pharmacy, The University of Hong Kong, Hong Kong SAR, China; ^6^Department of Anesthesiology, The Second Affiliated Hospital and Yuying Children's Hospital, Wenzhou Medical University, Wenzhou, China; ^7^Department of Anesthesiology, Affiliated Hospital of Guangdong Medical University, Zhanjiang, China

## Abstract

**Background:**

Constitutive nuclear factor kappa B (NF*κ*B) activation has been shown to exacerbate during myocardial ischemia/reperfusion (I/R) injury. We recently showed that miR-181c-5p exacerbated cardiomyocytes injury and apoptosis by directly targeting the 3′-untranslated region of protein tyrosine phosphatase nonreceptor type 4 (PTPN4). However, whether miR-181c-5p mediates cardiac I/R injury through NF*κ*B-mediated inflammation is unknown. Thus, the present study aimed to investigate the role of miR-181c-5p during myocardial I/R injury and explore its mechanism in relation to inflammation in H9C2 cardiomyocytes.

**Methods and Results:**

In hypoxia/reoxygenation (H/R, 6 h hypoxia followed by 6 h reoxygenation)-stimulated H9C2 cardiomyocytes or postischemic myocardium of rat, the expression of miR-181c-5p was significantly upregulated, which was concomitant increased NF*κ*B activity when compared to the nonhypoxic or nonischemic control groups. This is indicative that miR-181c-5p may be involved in NF*κ*B-mediated inflammation during myocardial I/R injury. To investigate the potential role of miR-181c-5p in H/R-induced cell inflammation and injury, H9C2 cardiomyocytes were transfected with the miR-181c-5p agomir. Overexpression of miR-181c-5p significantly aggravated H/R-induced cell injury (increased lactate dehydrogenase (LDH) level) and exacerbated NF*κ*B-mediated inflammation (greater phosphorylation and degradation of I*κ*B*α*, phosphorylation of p65, and increased levels of proinflammatory cytokines tumor necrosis factor *α* (TNF*α*), interleukin (IL)-6, and IL-1*β*). In contrast, inhibition of miR-181c-5p by its antagomir transfection *in vitro* had the opposite effect. Furthermore, overexpression of miR-181c-5p significantly enhanced lipopolysaccharide-induced NF*κ*B signalling. Additionally, knockdown of PTPN4, the direct target of miR-181c-5p, significantly aggravated H/R-induced phosphorylation and degradation of I*κ*B*α*, phosphorylation of p65, and the levels of proinflammatory cytokines. PTPN4 knockdown also cancelled miR-181c-5p antagomir mediated anti-inflammatory effects in H9C2 cardiomyocytes during H/R injury.

**Conclusions:**

It is concluded that miR-181c-5p may exacerbate myocardial I/R injury and NF*κ*B-mediated inflammation *via* PTPN4, and that targeting miR-181c-5p/PTPN4/NF*κ*B signalling may represent a novel strategy to combat myocardial I/R injury.

## 1. Introduction

Ischemic heart disease is one of the major causes of death worldwide [1, 2]. During cardiac ischemia/reperfusion (I/R) injury, cellular damage such as excessive apoptosis can result in protease and danger-associated molecular patterns that favours a proinflammatory environment through the activation of nuclear factor kappa B (NF*κ*B) [3]. In the ischemic heart, prolonged activation of NF*κ*B is generally considered to be detrimental by eliciting signals that trigger chronic inflammation through enhanced elaboration of proinflammatory cytokines, including interleukin (IL)-6, IL-1*β*, and tumor necrosis factor *α* (TNF*α*), leading to cardiac injury [4, 5]. Constitutive NF*κ*B activation has been demonstrated in various models of experimental myocardial ischemia and reperfusion [3, 6]. *In vivo* transfer of NF*κ*B decoy oligodeoxynucleotides to bind transcriptional factor, blocking inflammatory gene activation, reduced the extent of myocardial infarction following reperfusion [7]. Thus, a strategy that can inhibit excessive NF*κ*B-mediated inflammation should be an effective therapy to combat ischemic heart disease.

The miR-181 family (including miR-181a, miR-181b, miR-181c, and miR-181d) plays diverse roles in regulating various cellular and biological processes through posttranscriptional regulation of target genes [8–12]. Accumulating evidence suggests a central role for the miR-181 family in inflammation by regulating target proteins invovled in critical inflammatory signalling pathways, such as NF*κ*B signalling [13, 14]. For example, miR-181b can reduce inflammation through targeting the 3′-untranslated region of the importin-*α*3 (a protein critical for the translocation of NF*κ*B from the cytoplasm to the nucleus), further inhibiting the activation of NF*κ*B signalling pathway [14]. In addition, previous study has demonstrated that miR-181c-5p (named miR-181c in other studies) can directly target the 3′-untranslated region of TNF*α* mRNA, suppressing its mRNA and protein expression in rat microglial cells after ischemia injury [15]. However, the anti-inflammatory effect of miR-181c-5p in neuroinflammation was challenged as miR-181c-5p exacerbated brain injury in acute ischemic stroke [16]. Thus, it is still unclear whether miR-181c-5p exerts pro- or anti-inflammatory effect in the context of I/R in general, and in myocardial I/R injury in specific.

We have recently shown that miR-181c-5p exacerbated hypoxia/reoxygenation (H/R)-induced cardiomyocyte injury and apoptosis via targeting protein tyrosine phosphatase nonreceptor type 4 (PTPN4) [17]. Of note, PTPN4 suppresses Toll-like receptor 4/NF*κ*B signalling in mouse peritoneal macrophages [18]. However, it is unknown whether or not PTPN4 may inhibit NF*κ*B-mediated proinflammatory responses in cardiomyocytes. During myocardial I/R injury, the cardiac resident cells, such as cardiomyocytes may elaborate proinflammatory cytokines in response to various stimuli and thus favour a proinflammatory environment. Therefore, the present study aimed to determine whether or not miR-181c-5p enhances NF*κ*B-mediated inflammation via inhibiting PTPN4 during cardiac I/R injury by using rat origin cardiomyocytes (H9C2) subjected to H/R stimulation.

## 2. Materials and Methods

### 2.1. Cell Culture

The rat origin H9C2 cardiomyocytes were purchased from the American Type Culture Collection (ATCC, Manassas, VA, USA). The H9C2 cells were maintained in Dulbecco's Modified Eagle's Medium (DMEM, ThermoFisher Scientific, MA, USA) supplemented with 10% fetal bovine serum (FBS, Biosera, Kansas City, MO, USA) and 1% penicillin/streptomycin (100 U/ml, ThermoFisher Scientific). All cells were cultured in a humidified atmosphere containing 5% CO_2_-95%O_2_ at 37°C.

### 2.2. Cell Treatment

The H9C2 cardiomyocytes were seeded into six-well plate (2 × 10^5^ cell/well) overnight and transfected with micrON rno-miR-181c-5p agomir (50 nM, RIBOBIO, Guangzhou, China), micrOFF rno-miR-181c-5p antagomir (50 nM, RIBOBIO), small RNA (siRNA) against PTPN4 (50 nM, RIBOBIO), or their negative controls using Lipofectamine 2000 (Invitrogen, Carlsbad, CA, USA) for 24 h according to the manufacturer's instructions. The subgroups of these cardiomyocytes were subsequently subjected to H/R or lipopolysaccharide (LPS, 3 *μ*g/ml, 21 h, Sigma) stimulation before harvesting. H/R stimulation was achieved as previously described [19]. Briefly, the H9C2 cardiomyocytes were cultured with DMEM medium (no glucose or FBS) for 6 h in a humidified Plexiglas chamber containing 95% N_2_ and 5% CO_2_. The cells were then exposed to fresh culture medium and room air atmosphere containing 5% CO_2_ and 95% O_2_ for an additional 6 h to achieve reoxygenation. The transfection effects were verified by detecting the expression of miR-181c-5p, mRNA, or protein expression of PTPN4 through real-time polymerase chain reaction (PCR) or Western blotting.

### 2.3. Measurement of Lactate Dehydrogenase (LDH) Activity

The content of LDH, which was released in the culture medium, was measured by LDH cytotoxicity assay kit (Roche, Germany) according to the manufacturer's instructions.

### 2.4. *In Vivo* Left Anterior Descending Artery Ligation Model

All experimental procedures were approved by The University of Hong Kong Committee on the Use of Live Animals for Teaching and Research. Male adult Sprague-Dawley rats (8 weeks of age) were anesthetized with ketamine (100 mg/kg) and xylazine (10 mg/kg). The *in vivo* myocardial I/R injury model was induced by occluding the left anterior descending (LAD) artery with a 7-0 silk suture for 30 min followed by 2 h of reperfusion [20]. A sham operation was performed by passing a silk thread under the LAD without occlusion. Myocardial infarct size (IS) was measured by using Evans blue/TTC (1% 2, 3, 5-triphenyltetrazolium chloride) staining and expressed as a percentage of the area at risk (AAR). At the harvest time, the heart was quickly collected for further measurement of miRNA, mRNA, and protein expression.

### 2.5. Real-Time Polymerase Chain Reaction

Total RNA was extracted from H9C2 cardiomyocytes or rat heart tissues using RNAiso Plus (Takara, Japan) and reverse transcribed to cDNA with PrimeScript RT Master Mix kit (Takara), according to the manufacturer's instructions. For reverse transcription of miR-181c-5p or U6 (served as an internal reference), specific Bulge-Loop™ miRNA primers (Ribobio) were used instead of the random primers which were included in the PrimeScript RT Master Mix kit. Quantitative real-time PCR was performed with a SYBR green master mix (Takara) on an Applied Biosystems Prism 7000 sequence detection system (Applied Biosystems, Foster City, CA, USA) as previously described [21]. Gene-specific primers were as follows: rat IL-6 forward: 5′-ACTTCACAAGTCGGAGGCTT-3′, reverse: 5′-AGTGCATCATCGCTGTTCAT-3′; rat IL-1*β* forward: 5′-TACCTATGTCTTGCCCGTGGA-3′, reverse: 5′-ATCATCCCACGAGTCACAGAGG-3′; rat TNF*α* forward: 5′-TCTCAAAACTCGAGTGACAAGC-3′, reverse: 5′-GGTTGTCTTTGAGATCCATGC-3′; rat PTPN4 forward: 5′-CCCTCTTCCCCTGAAAAGTC-3′, reverse: 5′-TCATGGGTGTGTTCTGCAAT-3′; rat *β*-actin forward: 5′-AGGCCAACCGTGAAAAGATG-3′, reverse: 5′-ACCAGAGGCATACAGGGACAA-3′. Relative mRNA or miRNA levels were quantified by using the 2^-*ΔΔ*Ct^ method and normalized to those of *β*-actin or U6, respectively.

### 2.6. Western Blotting

H9C2 cardiomyocytes or frozen heart tissues were homogenized in lysis buffer (Sigma) supplemented with Protease inhibitor cocktail tablet (Roche) and Phosphatase inhibitor cocktail tablet (Roche). Equal protein amounts were loaded and separated by 10% sodium dodecyl sulfate polyacrylamide gel electrophoresis and transferred onto polyvinylidene difluoride membranes for immunoblot analysis as previously described [22]. Antibodies against I*κ*B*α* (1 : 1000), phospho-I*κ*B*α* (Ser^32/36^) (1 : 1000), p65 (1 : 1000), phospho-p65 (Ser^536^) (1 : 1000), GAPDH (1 : 1000), and *β*-tubulin (1 : 3000) were purchased from Cell Signaling Technology and used as primary antibodies. Primary antibody against PTPN4 antibody (1 : 1000) was purchased from Novus. Horseradish peroxidase-conjugated antimouse (1 : 3000) or antirabbit (1 : 3000) secondary antibodies were purchased from Cell Signaling Technology. The blots were visualized with Amersham™ ECL Western Blotting Detection Reagent (GE Healthcare) and subsequently exposed to X-ray film (Carestream, NY, USA). Image J software (National Institutes of Health, MD, USA) was used to quantify the optical densities of the immunoreactive bands.

### 2.7. Statistical Analysis

All data are presented as means ± standard error of means (S.E.M.). Comparison between groups was carried out by two-tailed unpaired Student's *t*-test, one-way ANOVA, or two-way ANOVA followed by Bonferroni post hoc test, where appropriate, using the GraphPad Prism 8.0 software (San Diego, CA, USA). In all comparisons, *P* value less than 0.05 was considered as statistically significant difference.

## 3. Results

### 3.1. miR-181c-5p Was Upregulated Concomitantly with Enhanced NF*κ*B Activity in Posthypoxic H9C2 Cardiomyocytes and Postischemic Myocardium of Rat

**Figure 1:**
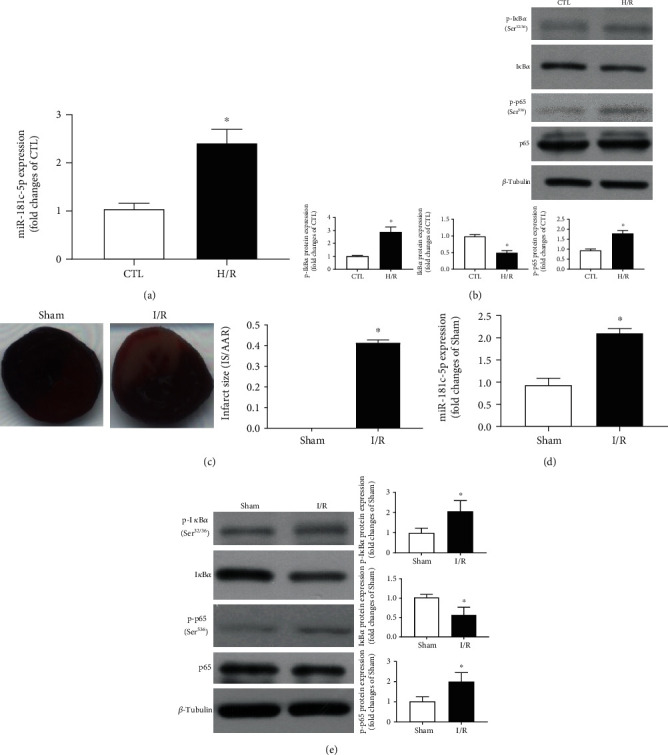
miR-181c-5p was upregulated concomitantly with enhanced NF*κ*B activity in posthypoxic H9C2 cardiomyocytes and postischemic myocardium of rat. (a) Increased expression of miR-181c-5p in hypoxia/reoxygenation (H/R, 6 h hypoxia followed by 6 h reoxygenation) stimulated H9C2 cardiomyocytes. (b) Representative Western blots of phosphorylated I*κ*B*α* (Ser^32/36^), I*κ*B*α*, phosphorylated p65 (Ser^526^), p65, and *β*-tubulin in the H/R-stimulated H9C2 cardiomyocytes. In the *in vivo* model, myocardial I/R (30 minutes of left anterior descending artery occlusion and 2 hours of reperfusion in rats) induced significant increased postischemic myocardial infarction size (c) and upregulation of miR-181c-5p (d). (e) Representative Western blots of phosphorylated I*κ*B*α* (Ser^32/36^), I*κ*B*α*, phosphorylated p65 (Ser^526^), p65, and *β*-tubulin in postischemic myocardium of rat. Protein presence of phosphorylated I*κ*B*α* (Ser^32/36^), I*κ*B*α*, and phosphorylated p65 (Ser^526^) was normalized to I*κ*B*α*, *β*-tubulin, and p65, respectively. Data are shown as means ± SEM; ∗*P* < 0.05 vs. CTL or Sham (two-tailed unpaired Student's *t*-test), *n* = 5.

We have previously reported that the expression of miR-181c-5p in H/R-stimulated H9C2 cardiomyocytes or postischemic myocardium of rat was significantly increased when compared to control groups [17]. Of note, the current study further demonstrated that upregulation of miR-181c-5p (Figure 1(a)) was paralleled by enhanced NF*κ*B activity, as evidenced by enhanced degradation of I*κ*B*α* and phosphorylation of I*κ*B*α* (Ser^32/36^) and p65 (Ser^536^) (Figure 1(b)) in H/R-stimulated H9C2 cardiomyocytes. Furthermore, in the *in vivo* myocardial I/R model, increased myocardial infarction size (Figure 1(c)) was accompanied by upregulation of miR-181c-5p (Figure 1(d)) and enhanced NF*κ*B activity (Figure 1(e)), suggesting that miR-181c-5p may be involved in the NF*κ*B-mediated proinflammatory responses of cardiomyocytes during the pathology of myocardial I/R injury.

### 3.2. Overexpression of miR-181c-5p Exacerbated NF*κ*B-Mediated Inflammation in H9C2 Cardiomyocytes in Response to H/R Stimulation

**Figure 2:**
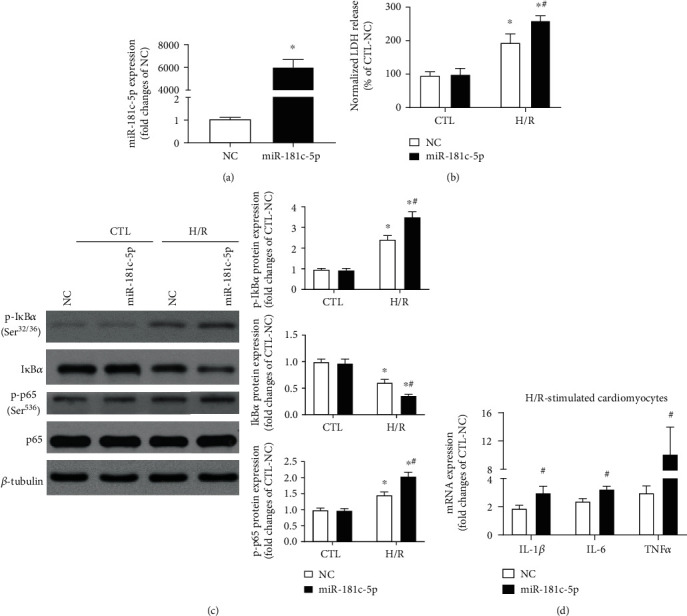
Overexpression of miR-181c-5p exacerbated NF*κ*B-mediated inflammation in H9C2 cardiomyocytes in response to H/R stimulation. miR-181c-5p agomir (miR-181c-5p) transfection resulted in significant overexpression of miR-181c-5p in H9C2 cardiomyocytes (a), and overexpression of miR-181c-5p exacerbated the H/R-induced LDH release (b). (c) Representative Western blots of phosphorylated I*κ*B*α* (Ser^32/36^), I*κ*B*α*, phosphorylated p65 (Ser^526^), p65, and *β*-tubulin in the NC- or miR-181c-5p agomir-transfected H9C2 cardiomyocytes with or without H/R stimulation. Protein presence of phosphorylated I*κ*B*α* (Ser^32/36^), I*κ*B*α*, and phosphorylated p65 (Ser^526^) was normalized to I*κ*B*α*, *β*-tubulin, and p65, respectively. (d) mRNA expression of NF*κ*B-dependent genes, including IL-1*β*, IL-6, and TNF*α* in the NC- or miR-181c-5p agomir-transfected H9C2 cardiomyocytes with H/R stimulation. mRNA levels are expressed as fold changes against those mRNA expressions in NC-transfected H9C2 cardiomyocytes with no stimulation. Data are shown as means ± SEM; ∗*P* < 0.05 vs. CTL, ^#^*P* < 0.05 vs. NC agomir (NC) (two-tailed unpaired Student's *t*-test in (a, d) and two-way ANOVA followed by Bonferroni test in (b, c)), *n* = 5.

To determine the role of miR-181c-5p in NF*κ*B-mediated inflammation in cardiomyocytes under hypoxic condition, the effect of miR-181c-5p on the key enzymes in the NF*κ*B signalling pathway and the expression of NF*κ*B-dependent genes were examined in H9C2 cardiomyocytes by using gain-of-function experiments. Overexpression of miR-181c-5p was achieved by transfection of miR-181c-5p agomir into H9C2 cardiomyocytes, which resulted in significant increases in the expression of miR-181c-5p (Figure 2(a)) and in the levels of H/R-stimulated release of LDH (Figure 2(b)), which were consistent with our previous report [17]. Overexpression of miR-181c-5p did not alter the presence of total p65 but significantly enhanced the degradation of I*κ*B*α* and increased the level of phosphorylated I*κ*B*α* (Ser^32/36^) and phosphorylated p65 (Ser^536^) in response to H/R stimulation (Figure 2(c)). Furthermore, the H/R-stimulated induction of NF*κ*B-mediated proinflammatory cytokines (including IL-1*β*, IL-6, and TNF*α*) was further increased in the presence of overexpression of miR-181c-5p by 58%, 72%, and 435%, respectively (Figure 2(d)). Taken in conjunction, these observations suggested that miR-181c-5p can exacerbate H/R-induced NF*κ*B signalling by facilitating the phosphorylation of I*κ*B*α* and p65, and thus lead to augmentation of NF*κ*B transcription activity.

### 3.3. Inhibition of miR-181c-5p Suppressed NF*κ*B-Mediated Inflammation in H9C2 Cardiomyocytes in Response to H/R Stimulation

**Figure 3:**
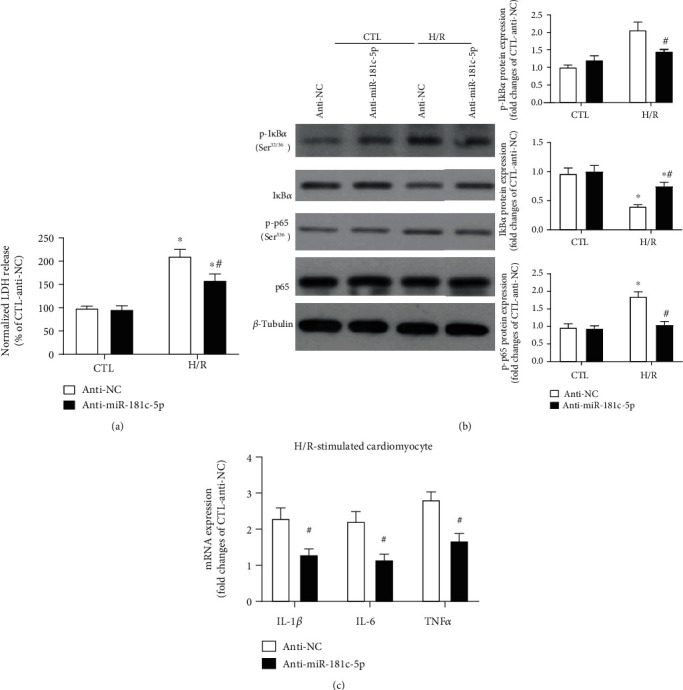
Inhibition of miR-181c-5p suppressed NF*κ*B-mediated inflammation in H9C2 cardiomyocytes in response to H/R stimulation. Inhibition of miR-181c-5p attenuated the H/R-induced LDH release (a). (b) Representative Western blots of phosphorylated I*κ*B*α* (Ser^32/36^), I*κ*B*α*, phosphorylated p65 (Ser^526^), p65, and *β*-tubulin in the anti-NC- or miR-181c-5p antagomir (anti-miR-181c-5p)-transfected H9C2 cardiomyocytes with or without H/R stimulation. Protein presence of phosphorylated I*κ*B*α* (Ser^32/36^), I*κ*B*α* and phosphorylated p65 (Ser^526^) was normalized to I*κ*B*α*, *β*-tubulin, and p65, respectively. (c) mRNA expression of NF*κ*B-dependent genes, including IL-1*β*, IL-6, and TNF*α* in the anti-NC- or anti-miR-181c-5p-transfected H9C2 cardiomyocytes with H/R stimulation. mRNA levels are expressed as fold changes against those mRNA expressions in anti-NC-transfected H9C2 cardiomyocytes with no stimulation. Anti-NC: negative control of miR-181c-5p antagomir; data are shown as means ± SEM; ∗*P* < 0.05 vs. CTL, ^#^*P* < 0.05 vs. NC antagomir (anti-NC) (two-way ANOVA followed by Bonferroni test in (a–c) and two-tailed unpaired Student's *t*-test in (d)), *n* = 5.

To consolidate the proinflammatory effect of miR-181c-5p on NF*κ*B signalling in cardiomyocytes, antinegative control (the negative control of miR-181c-5p antagomir) or miR-181c-5p antagomir was transfected into H9C2 cardiomyocytes and subsequently subjected to normoxia or H/R stimulation. As anticipated, inhibition of miR-181c-5p significantly attenuated the H/R-induced LDH leakage (Figure 3(a)). In normoxia group, there was a trend towards an increased expression of phosphorylation of I*κ*B*α* (Ser^32/36^) in miR-181c-5p antagomir-transfected H9C2 cells, which however did not reach statistical significance (Figure 3(b)). Upon H/R stimulation, inhibition of miR-181c-5p significantly suppressed the degradation of I*κ*B*α* and reduced the phosphorylation of I*κ*B*α* (Ser^32/36^) and p65 (Ser^536^) (Figure 3(b)). Moreover, inhibition of miR-181c-5p significantly suppressed the H/R-induced mRNA expression of NF*κ*B-mediated genes, including IL-1*β*, IL-6, and TNF*α* by 43%, 48%, and 40%, respectively (Figure 3(c)). Taken together, these findings indicated that inhibition of miR-181c-5p suppresses H/R-induced NF*κ*B signalling in H9C2 cardiomyocytes.

### 3.4. miR-181c-5p Exacerbated LPS-Induced NF*κ*B Signalling in H9C2 Cardiomyocytes

**Figure 4:**
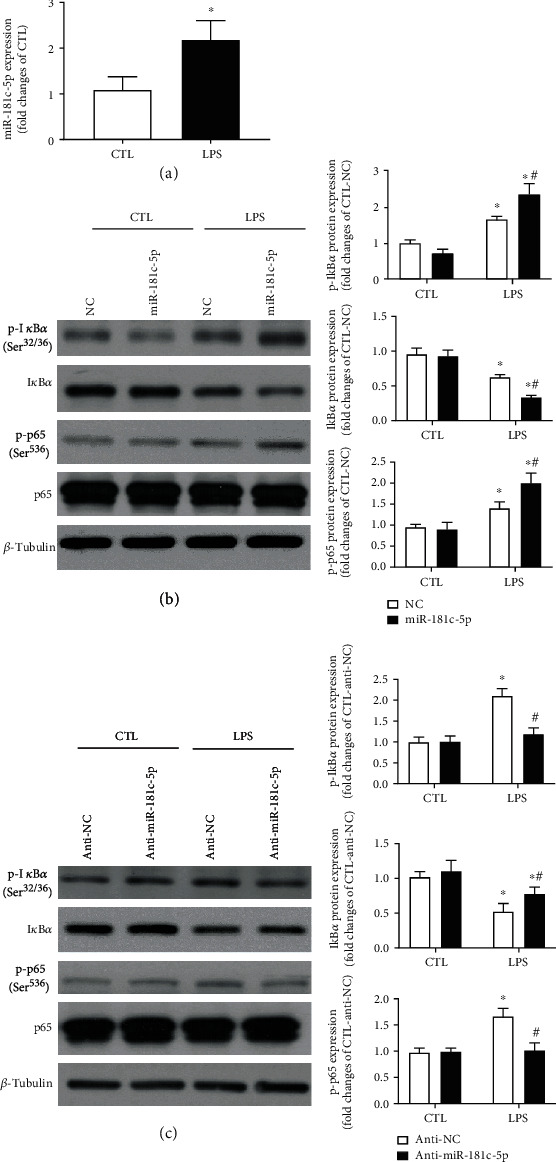
miR-181c-5p exacerbated LPS-induced NF*κ*B signalling in H9C2 cardiomyocytes. Increased expression of miR-181c-5p in lipopolysaccharide (LPS, 3 *μ*g/ml, 21 h) stimulated H9C2 cardiomyocytes (a). (b) Representative Western blots of phosphorylated I*κ*B*α* (Ser^32/36^), I*κ*B*α*, phosphorylated p65 (Ser^526^), p65, and *β*-tubulin in the NC- or miR-181c-5p agomir-transfected H9C2 cardiomyocytes with or without LPS stimulation. (c) Representative Western blots of phosphorylated I*κ*B*α* (Ser^32/36^), I*κ*B*α*, phosphorylated p65 (Ser^526^), p65, and *β*-tubulin in the anti-NC- or miR-181c-5p antagomir (anti-miR-181c-5p)-transfected H9C2 cardiomyocytes with or without LPS stimulation. Protein presence of phosphorylated I*κ*B*α* (Ser^32/36^), I*κ*B*α*, and phosphorylated p65 (Ser^526^) was normalized to I*κ*B*α*, *β*-tubulin, and p65, respectively. Anti-NC: negative control of miR-181c-5p antagomir; data are shown as means ± SEM; ∗*P* < 0.05 vs. CTL, ^#^*P* < 0.05 vs. NC agomir (NC) or NC antagomir (anti-NC) (two-tailed unpaired Student's *t*-test in (a) and two-way ANOVA followed by Bonferroni test in (b, c), *n* = 5.

To further explore whether or not miR-181c-5p could exacerbate NF*κ*B-mediated proinflammatory responses in cardiomyocytes, experiments were performed using another stimulus—LPS, which has been proved to induce NF*κ*B-dependent proinflammatory cytokines in cardiomyocytes [23, 24]. In response to LPS stimulation, the expression of miR-181c-5p was significantly increased to a level over 2-fold of that of unstimulated cells (Figure 4(a)), suggesting that miR-181c-5p may be involved in the proinflammatory responses of cardiomyocytes to LPS stimulation. In the unstimulated H9C2 cells, there was a trend towards a reduced phosphorylation of I*κ*B*α* (Ser^32/36^) in miR-181c-5p agomir-transfected H9C2 cells, which however did not reach statistical significance (Figure 4(b)). Furthermore, overexpression of miR-181c-5p significantly enhanced LPS-induced degradation of I*κ*B*α* and phosphorylation of I*κ*B*α* (Ser^32/36^) and p65 (Ser^536^) (Figure 4(b)). In contrast, inhibition of miR-181c-5p significantly attenuated LPS-induced degradation of I*κ*B*α* and phosphorylation of I*κ*B*α* (Ser^32/36^) and p65 (Ser^536^) (Figure 4(c)). Taken together, these results suggested that miR-181c-5p enhances NF*κ*B signalling in response to LPS stimulation in H9C2 cardiomyocytes.

### 3.5. Reduction of PTPN4 Mediated the Proinflammatory Effect of miR-181c-5p in H9C2 Cardiomyocytes

**Figure 5:**
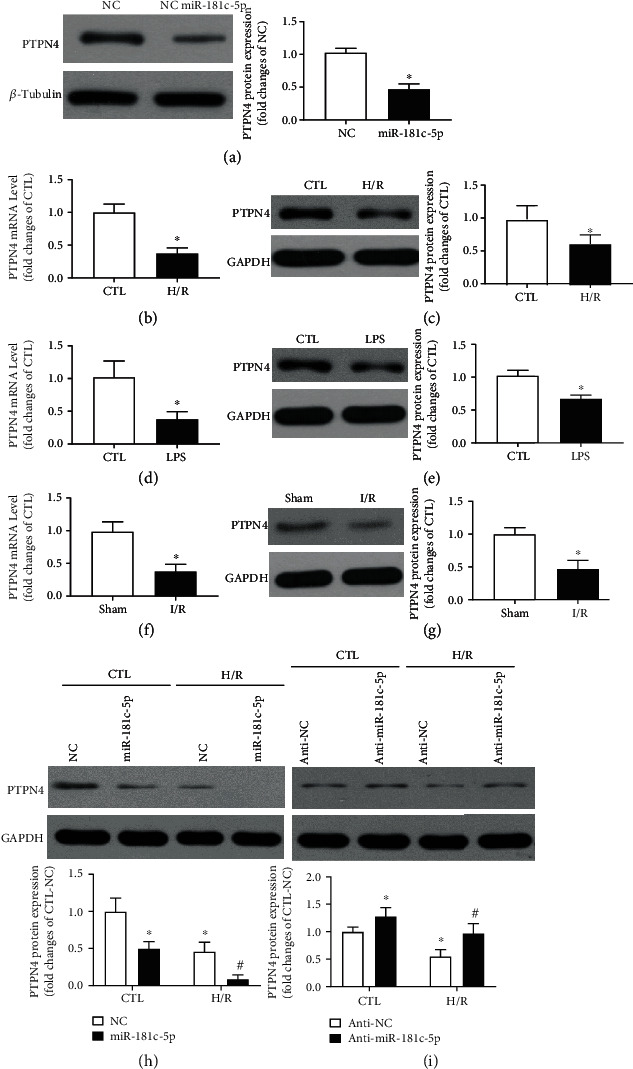
PTPN4 is the potential target of miR-181c-5p. Overexpression of miR-181c-5p results in reduced levels of protein expression (a) of PTPN4 in H9C2 cardiomyocytes. (b) mRNA and (c) protein expression in H9C2 cardiomyocytes with or without H/R stimulation. (d) mRNA and (e) protein expression in H9C2 cardiomyocytes with or without LPS stimulation. (f) mRNA and (g) protein expression in the postischemic myocardium of rat. Representative Western blots of PTPN4 and GAPDH in the miR-181c-5p agomir (h) or antagomir (i)-transfected H9C2 cardiomyocytes with or without H/R stimulation. Protein presence of PTPN4 was normalized to *β*-tubulin or GAPDH. mRNA levels are expressed as fold changes against the mRNA expression in H9C2 cardiomyocytes with no stimulation or myocardium in Sham group. Anti-NC: negative control of miR-181c-5p antagomir; data are shown as means ± SEM; ∗*P* < 0.05 vs. NC agomir (NC) vs. CTL or vs. Sham or vs. CTL-NC agomir (NC) or vs. CTL-NC antagomir (anti-NC), ^#^*P* < 0.05 vs. H/R-NC or vs. H/R- anti-NC (two-tailed unpaired Student's *t*-test in (a–g) and two-way ANOVA followed by Bonferroni test in (h, i)), *n* = 5.

We have previously reported that miR-181c-5p can directly bind to the 3′-untranslated region of PTPN4 [17]. Consistently, overexpression of miR-181c-5p leads to significant reduction of protein expression of PTPN4 in cardiomyocytes (Figure 5(a)). In addition, the expressions of PTPN4 mRNA and protein were both significantly reduced in the H/R or LPS-treated H9C2 cells when compared with unstimulated cells (Figures 5(b)–5(e)). Furthermore, the levels of PTPN4 mRNA and protein were also significantly suppressed in the postischemic myocardium of rat (Figures 5(f) and 5(g)), indicating that PTPN4 may be the potential target of miR-181c-5p. The protein level of PTPN4 was also measured in the H9C2 cells transfected with miR-181c-5p agomir or antagomir with or without H/R stimulation. Overexpression of miR-181c-5p led to significant reduction of PTPN4 at basal condition, and the PTPN4 expression was further reduced in miR-181c-5p agomir-transfected cells after H/R stimulation (Figure 5(h)). In contrast, inhibition of miR-181c-5p led to significant increment of PTPN4 in both basal and H/R-stimulated condition (Figure 5(i)). Taken together, these observations lend further support to the interpretation that PTPN4 is the downstream target of miR-181c-5p.

**Figure 6:**
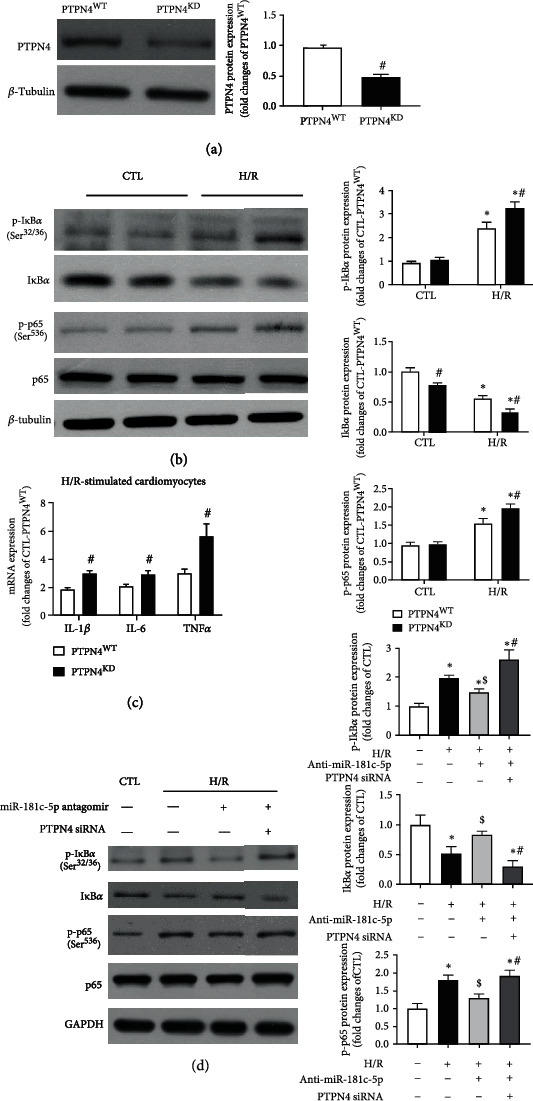
Reduction of PTPN4 mediated the proinflammatory effect of miR-181c-5p in H9C2 cardiomyocytes. (a) Transfection of cells with PTPN4 siRNA (PTPN4^KD^) resulted in significant reduction of PTPN4 protein level in H9C2 cardiomyocytes. (b) Representative Western blots of phosphorylated I*κ*B*α* (Ser^32/36^), I*κ*B*α*, phosphorylated p65 (Ser^526^), p65, and *β*-tubulin in the scramble siRNA or PTPN4 siRNA transfected H9C2 cardiomyocytes with or without H/R stimulation. (c) mRNA expression of NF*κ*B-dependent genes, including IL-1*β*, IL-6, and TNF*α* in the scramble siRNA or PTPN4 siRNA-transfected H9C2 cardiomyocytes with H/R stimulation. mRNA levels are expressed as fold changes against those mRNA expressions in scramble siRNA-transfected H9C2 cardiomyocytes with no stimulation. (d) Representative Western blots of phosphorylated I*κ*B*α* (Ser^32/36^), I*κ*B*α*, phosphorylated p65 (Ser^526^), p65, and GAPDH in the H9C2 cardiomyocytes cotransfected with miR-181c-5p antagomir and PTPN4 siRNA and subjected to H/R stimulation. Protein presence of phosphorylated I*κ*B*α* (Ser^32/36^), I*κ*B*α*, and phosphorylated p65 (Ser^526^) was normalized to I*κ*B*α*, *β*-tubulin/GAPDH, and p65, respectively. Data are shown as means ± SEM; ∗*P* < 0.05 vs. CTL or vs. NC agomir (NC) or no treatment group, ^#^*P* < 0.05 vs. PTPN4^WT^ or vs. H/R+miR-181c-5p antagomir, ^$^*P* < 0.05 vs. H/R (two-tailed unpaired Student's *t*-test in (a, c), two-way ANOVA followed by Bonferroni test in (b), and one-way ANOVA followed by Bonferroni test in (d)), *n* = 5.

Although emerging evidence has demonstrated that PTPN4 inhibits Toll-like receptor 4/NF*κ*B signalling in mouse peritoneal macrophages [18], it is still unclear whether or not PTPN4 suppresses NF*κ*B-mediated proinflammatory responses in cardiomyocytes, especially in the context of H/R-stimulation. To further explore whether PTPN4 knockdown can reproduce the proinflammatory effect of miR-181c-5p, PTPN4 knockdown model was established in H9C2 cardiomyocytes by using siRNA technology, and these cells were subsequently subjected to normoxia or H/R stimulation. Transfection of PTPN4 siRNA significantly reduced the endogenous protein levels (Figure 6(a)) of PTPN4 in H9C2 cells when compared with mock-transfected cells. As anticipated, PTPN4 knockdown significantly enhanced H/R-induced degradation of I*κ*B*α*, phosphorylation of I*κ*B*α* (Ser^32/36^) and p65 (Ser^536^) (Figure 6(b)), and mRNA expression of NF*κ*B-mediated proinflammatory cytokines (including IL-1*β*, IL-6, and TNF*α*) (Figure 6(c)). To strengthen the notion that reduction of PTPN4 mediates the proinflammatory effect of miR-181c-5p during H/R-induced cell injury, the H9C2 cells were cotransfected with miR-181c-5p antagomir and PTPN4 siRNA and subjected to H/R stimulation. Transfection of miR-181c-5p antagomir alone significantly attenuated phosphorylation of I*κ*B*α* (Ser^32/36^), degradation of I*κ*B*α*, and phosphorylation of p65 (Ser^536^) upon H/R stimulation, while cotransfection of miR-181c-5p antagomir and PTPN4 siRNA cancelled the anti-inflammatory effect of miR-181c-5p antagomir, as evidenced by enhanced phosphorylation of I*κ*B*α* (Ser^32/36^), degradation of I*κ*B*α*, and phosphorylation of p65 (Ser^536^) (Figure 6(d)). Collectively, these results demonstrated that miR-181c-5p may exacerbate NF*κ*B signalling pathway and thus aggravate cardiomyocyte inflammation and cell injury by directly targeting PTPN4 expression in H9C2 cardiomyocytes.

## 4. Discussion

Given the detrimental effect of the sustained NF*κ*B activation in the ischemic heart disease [5, 6], examining ways to attenuate excessive NF*κ*B-mediated inflammation is of clinical interest to combat cardiac I/R injury. The present study demonstrated that the NF*κ*B activity was significantly increased, with concomitantly upregulated miR-181c-5p level in the postischemic myocardium and H/R-stimulated H9C2 cardiomyocytes when compared to the control groups, suggesting that increased level of miR-181c-5p may be involved in the NF*κ*B-mediated inflammation during myocardial I/R injury. Indeed, overexpression of miR-181c-5p exacerbated H/R-induced cell injury (greater LDH leakage), and its proinflammatory effect in cardiomyocytes involves activation of NF*κ*B signalling pathway, as evidenced by enhanced degradation of I*κ*B*α*, increased level of phosphorylated I*κ*B*α* (Ser^32/36^) and phosphorylated p65 (Ser^536^), and augmented expression of proinflammatory cytokines in response to H/R stimulation. In contrast, inhibition of miR-181c-5p *in vitro* had the opposite effect in NF*κ*B-mediated inflammation. Of note, neither overexpression nor inhibition miR-181c-5p altered the phosphorylated I*κ*B*α* (Ser^32/36^) or total I*κ*B*α* at basal condition. The proinflammatory effect of miR-181c-5p may require the suppression or elevation of some other molecules during H/R. Indeed, in response to H/R, multiple signalling pathways were altered, such as hypoxia-inducible factor 1-*α* (HIF-1*α*) [20] and cyclooxygenase-2 (COX-2) [19], both of which are involved in the H/R-induced inflammation. However, whether HIF-1*α*, COX-2, or other molecules work as cofactors and participate in the proinflammatory effect of miR-181c-5p are still unclear and warrants further investigation. Taken together, these results indicated that miR-181c-5p enhances NF*κ*B-mediated inflammation in cardiomyocytes in response to H/R stimulation.

In addition to explore the role of miR-181c-5p in H/R stimulation induced inflammation, LPS was used in the present study as another stimulus to activate NF*κ*B signalling pathway in H9C2 cardiomyocytes. In response to LPS stimulation, I*κ*B*α* is phosphorylated at serine 32 and 36, followed by ubiquitination and proteasome-mediated degradation [25, 26], leading to the dissociation of I*κ*B*α* from NF*κ*B. The activated NF*κ*B moves into the nucleus and binds to specific sequences of DNA *κ*B sites resulting in the transcription of NF*κ*B-mediated genes [25, 26]. As a component of the bacterial cell wall, LPS has been widely used to establish sepsis model *in vivo* and *in vitro*, because LPS induces profound inflammation and pathological consequences similar to those found during sepsis [27, 28]. Sepsis, an acute inflammatory disease, is a life-threatening condition that follows bacterial infection [29, 30]. Cardiac dysfunction could be an important consequence of sepsis/septic shock and contributes to the high mortality because of the elevated inflammation [29, 30]. In the present study, overexpression of miR-181c-5p further enhanced LPS-induced NF*κ*B signalling, whereas inhibition of miR-181c-5p attenuated LPS-stimulated NF*κ*B activation. These observations not only consolidate the proinflammatory effect of miR-181c-5p in cardiomyocytes but also open an exciting research field to investigate the role of miR-181c-5p in cardiac dysfunction during the pathogenesis of sepsis and/or in during the likewise inflammation subsequent to myocardial I/R. To the best of our knowledge, the proinflammatory effect of miR-181c-5p in sepsis, especially sepsis-induced myocardial dysfunction has not been explored yet but is worth further investigation.

In response to different stimuli, besides to the classical phosphorylation sites of I*κ*B*α* at serine 32 and 36, phosphorylation of I*κ*B*α* at tyrosine residue 42 also mediates the degradation of I*κ*B*α* and the subsequent NF*κ*B activation [31, 32]. In a T lymphocytic cell line (EL4), mutation of Ser^32/36^ in I*κ*B*α* had no effect on H_2_O_2_-induced NF*κ*B activation, but mutation of tyrosine 42 abolished NF*κ*B activation by H_2_O_2_ [31]. Consistently, the cardiac NF*κ*B activation was completely blocked in a murine model which expressed the mutant I*κ*B*α* (S32A, S36A, Y42F) in a cardiac-specific manner, while in the mice expressed two mutant I*κ*B*α* (S32A, S36A), the NF*κ*B activation was only partially blocked (70-80%), indicating that phosphorylation of I*κ*B*α* at tyrosine residue 42 mediates NF*κ*B activation independent of Ser^32/36^ phosphorylation in I*κ*B*α* [32]. In addition, in HepG2 liver cells, in response to TNF*α*, activated cytosolic calpains has been shown to degrade I*κ*B*α* and activate NF*κ*B signalling independently of the ubiquitin-proteasome pathway [33]. In the present study, overexpression of miR-181c-5p leads to the phosphorylation of I*κ*B*α* at Serine 32 and 36, I*κ*B*α* degradation and NF*κ*B activation in H9C2 cells in response to H/R. However, whether or not miR-181c-5p may affect the phosphorylation of I*κ*B*α* at tyrosine residue 42 or cytosolic calpains activity is still unclear and merits further studies.

During the course of our study, we noticed that there are some studies reported that miR-181c-5p can attenuate excessive neuroinflammation through directly targeting the 3′-untranslated region of TNF*α* mRNA, suppressing its mRNA and protein expression in rat microglial cells after ischemia injury [15], which is opposite to our findings that miR-181c-5p exerts proinflammatory effect through enhanced NF*κ*B signalling in cardiomyocytes. The different cell types (BV-2 microglial cells vs. H9C2 cardiomyocytes) could be the main explanation regarding the discrepancy between our and others' study. The proinflammatory effect of miR-181c-5p may be cell specific. Similarly, the inhibitory effect of miR-181b (another member in miR-181 family) on NF*κ*B activation was specific to endothelial cells but not observed in other cell types, such as peripheral blood mononuclear cells [14]. Additionally, the expression pattern of miR-181c-5p varies in different cell types in response to stimulation. In oxygen-glucose deprivation activated BV-2 cells, there was significantly reduced level of miR-181c-5p in a time-dependent manner when compared with nonstimulated cells [15]. However, in the present study, in H9C2 cardiomyocytes, the expression of miR-181c-5p was significantly upregulated in response to H/R or LPS stimulation. Furthermore, in our study, transfection with miR-181c-5p agomir leads to an approximately 6000-fold increase of miR-181c-5p level, whereas there is only 20-fold increase of miR-181c-5p in others' work [15], which may also be viewed as a discrepancy between our and others' study. Moreover, the unaltered mRNA expression of TNF*α* in the H9C2 cardiomyocytes with miR-181c-5p overexpression also rules out the possibility that miR-181c-5p can directly target 3′-untranslated region of TNF*α* mRNA in the current experimental setting. In addition, loss of miR-181c-5p in the mitochondrial compartment shows cardioprotective effects during myocardial I/R injury [34]. In another study, miR-181c-5p aggravates brain injury in acute ischemic stroke through enhancement of apoptosis of microglia and neurons [16]. Taken together, these studies provide additional evidence that miR-181c-5p may play detrimental (proinflammatory) roles during ischemic attack.

We recently reported that miR-181c-5p exacerbates cardiomyocytes injury and apoptosis by directly targeting the 3′-untranslated region of PTPN4 [17]. This target was substantiated through several lines of evidence: (1) overexpression of miR-181c-5p results in the significantly reduction of protein level of PTPN4 in H9C2 cardiomyocytes, which has also been repeated in the present study; (2) significant reduction of mRNA and protein level of PTPN4 in H9C2 in H/R or LPS-treated H9C2 cardiomyocytes and postischemic myocardium of rat; (3) complementary sequence of miRNA-181c-5p was located on the positions from 4915 to 4921 or from 6333 to 6339 (or both) on the 3′UTR of rat PTPN4 mRNA, and there were 7 pairs of Watson-Crisk match; (4) mutation of miR-181c-5p binding sites blocked miR-181c-5p-mediated repression of PTPN4 in 293T cells [17]; (5) PTPN4 knockdown recapitulated the proapoptotic effect of miR-181c-5p in H9C2 cardiomyocytes. In addition to its reported effects in protecting against cell apoptosis, PTPN4 has been also reported to suppress Toll-like receptor 4 and may inhibit its downstream NF*κ*B signalling in mouse peritoneal macrophages [18]. These findings prompted us to hypothesize that miR-181c-5p may enhance NF*κ*B-mediated inflammation through targeting PTPN4 in H9C2 cardiomyocytes. In line with this speculation, siRNA-mediated knockdown of PTPN4 expression reproduced the proinflammatory effect of miR-181c-5p on NF*κ*B signalling in cardiomyocytes, as evidenced by the increased H/R-induced degradation of I*κ*B*α*, phosphorylation of I*κ*B*α* (Ser^32/36^) and p65 (Ser^536^), and mRNA expression of NF*κ*B-mediated proinflammatory cytokines (including IL-6, IL-1*β*, and TNF*α*]. Furthermore, cotransfection with miR-181c-5p antagomir and PTPN4 siRNA cancelled the anti-inflammatory effect of miR-181c-5p antagomir. Taken in conjunction, these findings implicate that miR-181c-5p may serve as a regulator of NF*κ*B-mediated inflammation through targeting PTPN4 in H9C2 cardiomyocytes. It is still unclear whether or not miR-181c-5p targets PTPN4 and exerts proinflammatory effect *in vivo* during I/R injury and warrants investigation in the future study by using miR-181c-5p knockout/overexpression mice.

## 5. Conclusion

In conclusion, the present study demonstrates that miR-181c-5p is involved in the enhanced NF*κ*B-mediated inflammation through targeting PTPN4 during myocardial I/R injury or H/R-stimulated cardiomyocyte injury. These observations suggest that increased miR-181c-5p level may serve as a potential risk factor, and future studies will focus on the potential clinical use of miR-181c-5p to combat inflammatory diseases, including ischemic heart disease.

## Data Availability

The data used to support the findings of this study are available from the corresponding author upon request.

## References

[1] Elgendy I. Y., Mahtta D., Pepine C. J. (2019). Medical therapy for heart failure caused by ischemic heart disease. *Circulation Research*.

[2] Pang L., Cai Y., Tang E. H. C., Irwin M. G., Ma H., Xia Z. (2016). Prostaglandin E receptor subtype 4 signaling in the heart: role in ischemia/reperfusion injury and cardiac hypertrophy. *Journal Diabetes Research*.

[3] Kim J. W., Jin Y. C., Kim Y. M. (2009). Daidzein administration *in vivo* reduces myocardial injury in a rat ischemia/reperfusion model by inhibiting NF-kB activation. *Life Sciences*.

[4] Dhingra R., Shaw J. A., Aviv Y., Kirshenbaum L. A. (2010). Dichotomous actions of NF-kappaB signaling pathways in heart. *Journal of Cardiovascular Translational Research*.

[5] Gordon J. W., Shaw J. A., Kirshenbaum L. A. (2011). Multiple facets of NF-*κ*B in the heart: to be or not to NF-*κ*B. *Circulation Research*.

[6] Jin Y. C., Kim C. W., Kim Y. M. (2009). Cryptotanshinone, a lipophilic compound of *Salvia miltiorrriza* root, inhibits TNF-*α*-induced expression of adhesion molecules in HUVEC and attenuates rat myocardial ischemia/reperfusion injury *in vivo*. *European Journal of Pharmacology*.

[7] Morishita R., Sugimoto T., Aoki M. (1997). *In vivo* transfection of *cis* element "decoy" against nuclear factor- *κ*B binding site prevents myocardial infarction. *Nature Medicine*.

[8] Jiang Y., Gong Y., Lin N., Qiu W. (2019). Expression of miR‑181a and TGF‑*β*2 in lens epithelial cells of patients with cataractous retinal detachment and its clinical significance. *Experimental and Therapeutic Medicine*.

[9] Li F. J., Zhang C. L., Luo X. J., Peng J., Yang T. L. (2019). Involvement of the MiR-181b-5p/HMGB1 pathway in Ang II-induced phenotypic transformation of smooth muscle cells in hypertension. *Aging and Disease*.

[10] Ai J., Gong C., Wu J. (2019). MicroRNA-181c suppresses growth and metastasis of hepatocellular carcinoma by modulating NCAPG. *Cancer Management and Research*.

[11] Wang H., Li J., Chi H. (2015). MicroRNA-181c targets Bcl-2 and regulates mitochondrial morphology in myocardial cells. *Journal of Cellular and Molecular Medicine*.

[12] Fan K. L., Li M. F., Cui F. (2019). Altered exosomal miR-181d and miR-30a related to the pathogenesis of CVB3 induced myocarditis by targeting SOCS3. *European Review for Medical and Pharmacological Sciences*.

[13] Sun X., Sit A., Feinberg M. W. (2014). Role of miR-181 family in regulating vascular inflammation and immunity. *Trends in Cardiovascular Medicine*.

[14] Sun X., Icli B., Wara A. K. (2012). MicroRNA-181b regulates NF-*κ*B-mediated vascular inflammation. *The Journal of Clinical Investigation*.

[15] Zhang L., Dong L. Y., Li Y. J., Hong Z., Wei W. S. (2012). The microRNA miR-181c controls microglia-mediated neuronal apoptosis by suppressing tumor necrosis factor. *Journal of Neuroinflammation*.

[16] Ma Q., Zhao H., Tao Z. (2016). MicroRNA-181c exacerbates brain injury in acute ischemic stroke. *Aging and Disease*.

[17] Ge L., Cai Y., Ying F. (2019). miR-181c-5p exacerbates hypoxia/reoxygenation-induced cardiomyocyte apoptosis via targeting PTPN4. *Oxidative Medicine and Cellular Longevity*.

[18] Huai W., Song H., Wang L. (2015). Phosphatase PTPN4 preferentially inhibits TRIF-dependent TLR4 pathway by dephosphorylating TRAM. *Journal of Immunology*.

[19] Pang L., Cai Y., Tang E. H. C. (2016). Cox-2 inhibition protects against hypoxia/reoxygenation-induced cardiomyocyte apoptosis via Akt-dependent enhancement of iNOS expression. *Oxidative Medicine and Cellular Longevity*.

[20] Mao X., Wang T., Liu Y. (2013). N-acetylcysteine and allopurinol confer synergy in attenuating myocardial ischemia injury via restoring HIF-1*α*/HO-1 signaling in diabetic rats. *PLoS One*.

[21] Cai Y., Ying F., Song E. (2015). Mice lacking prostaglandin E receptor subtype 4 manifest disrupted lipid metabolism attributable to impaired triglyceride clearance. *The FASEB Journal*.

[22] Cai Y., Ying F., Liu H. (2020). Deletion of Rap1 protects against myocardial ischemia/reperfusion injury through suppressing cell apoptosis via activation of STAT3 signaling. *The FASEB Journal*.

[23] Ren Q., Zhao S., Ren C., Ma Z. (2018). Astragalus polysaccharide alleviates LPS-induced inflammation injury by regulating miR-127 in H9c2 cardiomyoblasts. *International Journal of Immunopathology and Pharmacology*.

[24] Franceschelli S., Pesce M., Ferrone A. (2017). Biological effect of licochalcone C on the regulation of PI3K/Akt/eNOS and NF-*κ*B/iNOS/NO signaling pathways in H9c2 cells in response to LPS stimulation. *International Journal of Molecular Sciences*.

[25] Cai Y., Sukhova G. K., Wong H. K. (2015). Rap1 induces cytokine production in pro-inflammatory macrophages through NF*κ*B signaling and is highly expressed in human atherosclerotic lesions. *Cell Cycle*.

[26] Ghosh S., May M. J., Kopp E. B. (1998). NF-*κ*B and Rel proteins: evolutionarily conserved mediators of immune responses. *Annual Review of Immunology*.

[27] Gao M., Wang X., Zhang X. (2015). Attenuation of cardiac dysfunction in polymicrobial sepsis by MicroRNA-146a is mediated via targeting of IRAK1 and TRAF6 expression. *Journal of Immunology*.

[28] Drosatos K., Lymperopoulos A., Kennel P. J., Pollak N., Schulze P. C., Goldberg I. J. (2015). Pathophysiology of sepsis-related cardiac dysfunction: driven by inflammation, energy mismanagement, or both?. *Current Heart Failure Reports*.

[29] Merx M. W., Weber C. (2007). Sepsis and the heart. *Circulation*.

[30] Fattahi F., Ward P. A. (2017). Complement and sepsis-induced heart dysfunction. *Molecular Immunology*.

[31] Schoonbroodt S., Ferreira V., Best-Belpomme M. (2000). Crucial role of the amino-terminal tyrosine residue 42 and the carboxyl-terminal PEST domain of I*κ*B*α* in NF-*κ*B activation by an oxidative stress. *Journal of Immunology*.

[32] Brown M., McGuinness M., Wright T. (2005). Cardiac-specific blockade of NF-kappaB in cardiac pathophysiology: differences between acute and chronic stimuli in vivo. *American Journal of Physiology-Heart and Circulatory Physiology*.

[33] Han Y., Weinman S., Boldogh I., Walker R. K., Brasier A. R. (1999). Tumor necrosis Factor-*α*-inducible I*κ*B*α* proteolysis mediated by cytosolic m-Calpain. *The Journal of Biological Chemistry*.

[34] Das S., Kohr M., Dunkerly-Eyring B. (2017). Divergent effects of miR-181 family members on myocardial function through protective cytosolic and detrimental mitochondrial microRNA targets. *Journal of the American Heart Association*.

